# Neck circumference is associated with metabolic syndrome parameters in adolescents with obesity: a cross-sectional study

**DOI:** 10.1186/s12887-026-06949-6

**Published:** 2026-05-06

**Authors:** Mehmet Cengiz

**Affiliations:** Department of Pediatrics, Özel Gürlife Hospital, Fevzi Çakmak Mah. Akınsel Sok. No:1 Tepebaşı , Eskişehir, Türkiye

**Keywords:** Neck circumference, Metabolic syndrome, Adolescent obesity, Pediatric anthropometry, Insulin resistance, Pediatric siMS score

## Abstract

**Background:**

Neck circumference (NC) is a practical anthropometric measure for assessing obesity in youth, but its association with metabolic syndrome (MetS) components remains underexplored. This study evaluated the relationship between NC and MetS parameters in adolescents with obesity, using both standard International Diabetes Federation (IDF) criteria and expanded definitions incorporating insulin resistance.

**Methods:**

In this cross-sectional study, 48 adolescents with obesity and 41 age- and sex-matched controls were recruited from a tertiary pediatric clinic. Anthropometric data (BMI, waist circumference, NC) and fasting blood samples (glucose, insulin, HDL cholesterol, triglycerides) were collected. MetS was diagnosed using IDF criteria and an expanded definition including hyperinsulinemia or elevated HOMA-IR. The Pediatric siMS (PsiMS) score quantified metabolic risk.

**Results:**

NC was significantly greater in the obesity group than in controls (mean ± SD: 37.95 ± 3.03 cm vs. 31.86 ± 3.30 cm, *p* < .001). MetS prevalence was 14.6% by IDF criteria, rising to 43.8% with expanded criteria. In adolescents with obesity, NC positively correlated with fasting insulin, HOMA-IR, triglycerides, systolic blood pressure, and PsiMS score (*p* < .05 for all). NC was also associated with the total number of MetS criteria met under both diagnostic approaches.

**Conclusions:**

NC is a reliable, non-invasive marker correlated with key metabolic risk factors in adolescents with obesity. Its routine use could aid early detection of metabolic risk, particularly in resource-limited settings. Longitudinal research is needed to define NC thresholds and its predictive value for long-term outcomes.

## Introduction

Obesity has emerged as a major global public health challenge and is now widely recognized as a pandemic [1]. In children and adolescents, obesity is defined as a body mass index (BMI) at or above the 95th percentile for age and sex. The prevalence of obesity among individuals aged 2–19 years is approximately 17%, meaning that about 17% of adolescents have a BMI above the 95th percentile of historical reference populations [2]. Children who are overweight or obese are more likely to remain so during adolescence and adulthood. Furthermore, obesity during childhood and adolescence has been associated with an increased risk of adult-onset cardiovascular disease, stroke, type 2 diabetes, and certain types of cancer [3].

Traditionally, overall adiposity has been assessed using BMI, while abdominal fat distribution is typically evaluated through waist circumference (WC). These anthropometric parameters serve as important indicators of obesity-related health risks. However, BMI may not always be the most accurate measure of adiposity. Although it is routinely calculated from weight and height—often automatically in clinical settings with electronic health records—it requires access to both a scale and a stadiometer. In contrast, neck circumference (NC) can be obtained rapidly using only a measuring tape, making it an easier and more practical anthropometric tool for screening in diverse clinical and field settings [4]. In recent years, NC has gained attention as a simpler and more practical anthropometric marker for assessing obesity [5].

According to a comprehensive meta-analysis by Ma et al. [6], the diagnostic performance of NC for detecting overweight and obesity in children and adolescents was moderate, with pooled sensitivity and specificity values of 0.78 and 0.75, respectively. While NC demonstrates slightly lower diagnostic accuracy compared with BMI and WC percentile-based measures, it remains a practical and reliable anthropometric indicator—particularly advantageous in clinical and community settings where resources or time are limited. Initially used to identify individuals at risk for obstructive sleep apnea, NC has since been proposed as a useful tool for detecting overweight and obesity [6, 7].

A 2009 study conducted in Turkey highlighted the reliability and ease of NC measurement in identifying children with overweight or obesity [8] and a subsequent meta-analysis confirmed its moderate diagnostic accuracy for detecting excess weight in pediatric populations [6].

Metabolic syndrome (MetS), characterized by visceral obesity, systemic inflammation, and cellular dysfunction—often secondary to insulin resistance—is a well-established risk factor for adverse cardiovascular outcomes [9]. Studies have shown that children and adolescents with obesity have a significantly higher prevalence of MetS compared to their normal-weight peers [10]. While diagnostic criteria for MetS are well established in adults, there remains no universal consensus for the pediatric population. Among the available definitions, the criteria proposed by the International Diabetes Federation (IDF) are the most widely used in children and adolescents [10, 11]. In addition, expanded diagnostic criteria and scoring systems such as the pediatric siMS score have been developed for easier identification and quantification of metabolic risk [12]. It has been reported that the vast majority of children and adolescents with obesity meet at least one diagnostic criterion for MetS [13]. Given the rising prevalence of adolescent obesity, there is growing concern over the increased risk of early-onset type 2 diabetes and cardiovascular disease [14, 15]. Early detection and management of MetS are critical for mitigating the clinical progression of these conditions and reducing the long-term economic burden on healthcare systems [16, 17].

Several studies have explored the relationship between NC and MetS parameters in both adults and adolescents with obesity; however, limited research has focused on its diagnostic utility in adolescents using both conventional and expanded definitions of metabolic syndrome [18, 19]. To date, however, limited research has focused on the diagnostic utility of NC in adolescents using both conventional and expanded definitions of MetS and the use of NC as a practical and emerging screening tool in this context remains underexplored. Therefore, this study aimed to evaluate the association between neck circumference and metabolic syndrome criteria in adolescents with obesity.

## Methods

### Participants

This cross-sectional study was conducted between April 15 and July 15, 2022, among adolescents aged 11 to 18 years who presented to the Pediatric Outpatient Clinics at the University of Health Sciences Gülhane Training and Research Hospital in Ankara, Türkiye. Participants were recruited voluntarily and were included in either the obesity group or the control group based on BMI-for-age percentiles defined by national reference standards.

Adolescents were included in the obesity group if their BMI was at or above the 95th percentile for age and sex. Additional *inclusion criteria* were: no known chromosomal abnormalities, congenital metabolic or genetic disorders, and no use of medications that could influence blood glucose, lipid metabolism, blood pressure, or hormonal levels.

For the control group, participants were recruited from adolescents attending the pediatric outpatient clinic primarily for routine health evaluations, growth and weight monitoring, or non-endocrine minor complaints (such as nutritional counseling or routine follow-up visits), rather than for metabolic or endocrine disorders. The control group included adolescents whose BMI-for-age was between the 5th and 85th percentiles, corresponding to normal weight according to national reference standards. Underweight participants (BMI-for-age <5th percentile) were not included in the study.

### Sample size calculation

The minimum required sample size was calculated a priori using the G*Power 3.1 software. Based on previous studies examining correlations between neck circumference and metabolic parameters in pediatric populations, we assumed a medium effect size (*r* = .35), a significance level (α) of 0.05, and a power (1 − β) of 0.80. This analysis indicated that a minimum of 62 participants (31 per group) would be sufficient to detect a statistically significant association. To account for potential exclusions and missing data, 89 participants (48 with obesity and 41 controls) were ultimately included in the study.

The final number of participants in each group (48 with obesity and 41 controls) reflected the voluntary participation rate during the recruitment period. Although the target sample size was balanced between groups, slightly fewer control participants met the inclusion criteria and consented to participate. This difference did not affect statistical power, as both groups exceeded the minimum required sample size determined by a priori power analysis.

Ultimately, 48 adolescents were included in the obesity group and 41 in the control group.

#### Procedures and measurements

All anthropometric measurements were performed by the same trained clinician. Anthropometric measurements were obtained using standardized and calibrated equipment: body weight was measured with a digital electronic scale (SECA 799, Hamburg, Germany) to the nearest 0.1 kg, height was measured using a wall-mounted stadiometer (SECA 220, Hamburg, Germany) to the nearest 0.1 cm, and waist and neck circumferences were measured with a non-stretchable flexible tape (SECA 201, Hamburg, Germany). Neck circumference(NC) was measured with the participant standing erect, eyes facing forward, and shoulders relaxed. The measurement was taken at the level just below the laryngeal prominence (Adam’s apple) and perpendicular to the long axis of the neck using a non-stretchable flexible tape (SECA 201, Hamburg, Germany). The tape was applied snugly but without compression of the skin, and all measurements were taken twice by the same trained clinician; the mean value of the two readings was used for analysis. This standardized approach followed previously published pediatric anthropometric protocols [20].

Blood pressure (BP) was measured using an appropriately sized cuff (Welch Allyn Connex ProBP 3400, USA) with the participant seated and at rest for at least five minutes. Two consecutive measurements were obtained at one-minute intervals on the right arm, and the mean of the two readings was recorded for analysis. Measurements were performed in a quiet room by the same trained clinician to minimize inter-observer variability.

Following physical examination, fasting venous blood samples were collected to assess fasting plasma glucose, fasting insulin, high-density lipoprotein (HDL) cholesterol, and triglyceride levels. Participation was voluntary, and all data were anonymized and stored in a secure, locked cabinet to ensure confidentiality.

#### Definitions

Metabolic syndrome was defined based on the International Diabetes Federation (IDF) pediatric criteria, which include the presence of abdominal obesity (WC ≥ 90th percentile for age and sex or ≥ 80 cm in females and ≥ 94 cm in males), plus at least two of the following:

1. Fasting plasma glucose > 100 mg/dL or diagnosis of type 2 diabetes,
2. Low HDL cholesterol (< 40 mg/dL for ages 10–16 years; <40 mg/dL for males and < 50 mg/dL for females aged 16–18),
3. Elevated fasting triglycerides (≥ 150 mg/dL),
4. Elevated blood pressure (systolic ≥ 130 mmHg or diastolic ≥ 85 mmHg) [21].

In addition to the IDF criteria, we applied expanded criteria (termed “extended MetS” in this study) to enhance early detection of metabolic risk. Based on recent literature, elevated fasting insulin levels and insulin resistance (as measured by the HOMA-IR index) are considered early markers of impaired glucose metabolism [22]. Accordingly, impaired glucose regulation was defined as follows:

- For participants aged 10–16 years: fasting insulin ≥ 30 µU/mL or HOMA-IR > 3.82 (girls) or > 5.22 (boys).
- For participants aged ≥ 16 years: fasting insulin ≥ 20 µU/mL or HOMA-IR ≥ 3 [23–25].

In the extended definition, elevated blood pressure was defined as systolic or diastolic pressure ≥ 90th percentile for age, sex, and height, consistent with current pediatric guidelines [26].

Waist circumference (WC) percentiles were determined according to the age- and sex-specific Turkish reference data developed by Hatipoğlu et al. [27]. Abdominal obesity was defined as WC at or above the 90th percentile based on these national standards, consistent with the International Diabetes Federation (IDF) pediatric criteria.

Body mass index (BMI) was calculated as weight (kg) divided by height squared (m²) and expressed both as a raw value and as an age- and sex-adjusted BMI percentile national reference growth charts developed by Neyzi et al. for Turkish children and adolescents [28] which are widely used for assessing age- and sex-specific BMI percentiles in the Turkish population. While raw BMI values were used in descriptive statistics for comparison with other anthropometric measures, correlation analyses were conducted using BMI-for-age percentiles to account for age- and sex-related variation.

### Statistical analysis

Data were analyzed using IBM SPSS Statistics version 22. Descriptive statistics were reported as means (or mean ranks) for continuous variables and frequencies (percentages) for categorical variables. Normality of distribution for continuous variables was assessed using the Shapiro-Wilk and Kolmogorov-Smirnov tests, as well as evaluation of skewness and kurtosis. Since the data did not follow a normal distribution, nonparametric tests were used.

Because the distribution of continuous variables was non-normal, descriptive data were expressed as median and interquartile range (IQR), while categorical data were summarized as frequencies and percentages. Mean and standard deviation values were reported only for normally distributed demographic variables, such as age. As most continuous variables did not follow a normal distribution, data were summarized as median and interquartile range (IQR, 25th–75th percentile), and group comparisons were performed using the Mann–Whitney U test, and associations between categorical variables were analyzed with the Chi-square test.

Spearman’s correlation analysis was conducted to examine bivariate relationships between anthropometric and metabolic parameters. A p-value of < 0.05 was considered statistically significant.

To further evaluate the independent association between neck circumference (NC) and metabolic parameters, a multivariate linear regression analysis was performed. Age and sex were included as covariates to control for potential confounding. Although Tanner stage was not assessed, age was included as a covariate in the multivariate analyses to partially account for pubertal differences in body composition and fat distribution. Variables that demonstrated significant correlations with NC in univariate analyses (insulin, HOMA–IR, triglycerides, systolic blood pressure, and PsiMS score) were entered as dependent variables in separate models. The assumptions of multicollinearity and residual normality were checked and met.

### Ethics

The study protocol was reviewed and approved by the Clinical Research Ethics Committee of the Ministry of Health, Gülhane Training and Research Hospital (Approval Date: 11 May 2022; Approval Number: 2022/52). Written informed consent was obtained from all participants and/or their legal guardians prior to participation.

## Results

### Descriptive characteristics of the study and control groups

A total of 48 adolescents with obesity (54.2% female) and 41 adolescents in the control group (53.7% female) were included in the analysis. The mean age was 14.69 years (range: 11.04–17.99) in the obesity group and 14.45 years (range: 11.10–17.95) in the control group. There were no statistically significant differences in age or sex distribution between the groups.

Neck circumference (NC) ranged from 33.00 to 46.00 cm in the obesity group (mean ± SD: 37.95 ± 3.03 cm) and from 26.00 to 42.00 cm in the control group (mean ± SD: 31.86 ± 3.30 cm). Waist circumference (WC) ranged from 77.00 to 130.00 cm (mean: 103.40 ± 11.05 cm) in the obesity group and 56.00 to 92.00 cm (mean: 70.49 ± 9.61 cm) in controls.

When stratified by sex, neck circumference (NC) values were slightly higher in boys than in girls within both the obesity and control groups; however, these differences did not reach statistical significance (*p* > .05). Therefore, NC was not adjusted for sex in the correlation analyses.

Adolescents with obesity had significantly higher body weight, BMI, WC, and NC compared to controls (all *p* < .05). They also demonstrated significantly elevated fasting insulin levels, HOMA-IR index scores, serum triglyceride levels, and systolic blood pressure (all *p* < .05). In contrast, there were no significant group differences in fasting blood glucose or diastolic blood pressure (see Table 1).

**Table 1:** Descriptive findings and comparison of the study group and control group

	Study group (*n* = 48)	Control group (*n* = 41)	Statistics
Gender (females)	26 (54.2)	22 (53.7)	X^2^ = 0.00, *p* = .96^a^
Age (years)	14.68 (46.04)	14.45 (43.78)	Z = − 0.41, *p* = .68^b^
Body weight (kilograms)	88.23 (64.35)	50.94 (22.34)	Z = -7.65, ***p*** **= .00**^**b**^
Body mass index (BMI)	31.25 (65.50)	19.18 (21.00)	Z = -8.10, ***p*** **= .00**^**b**^
Waist circumference (cm)	103.40 (65.08)	70.49 (21.49)	Z = -7.94, ***p*** **= .00**^**b**^
Neck circumference (cm)	37.95 (62.05)	31.86 (25.04)	Z = -6.76, ***p*** **= .00**^**b**^
Metabolic parameters			
Fasting glucose (mg/dL)	81.02 (47.88)	78.48 (41.63)	Z = -1.14, *p* = .26^b^
Fasting insulin (µU/mL)	19.78 (60.78)	7.78 (26.52)	Z = -6.24, ***p*** **= .00**^**b**^
HOMA – IR (unitless index)	3.98 (60.52)	1.52 (26.83)	Z = -6.13, ***p*** **= .00**^**b**^
Triglycerides (mg/dL)	122.50 (52.78)	87.15 (35.89)	Z = -3.08, ***p*** **= .00**^**b**^
HDL cholesterol (mg/dL)	44.94 (37.38)	51.07 (53.93)	Z = -3.02, ***p*** **= .00**^**b**^
Systolic blood pressure (mmHg)	116.90	108.32	Z = -3.63, ***p*** **= .00**^**b**^
Diastolic blood pressure (mmHg)	71.94	69.66	Z = -1.04, *p* = .30^b^

Statistically significant *p* values are shown in bold color (*p* <.05). Data are presented as median (IQR) unless otherwise indicated. Categorical variables are expressed as frequencies and percentages. All biochemical parameters are expressed in mg/dL, insulin in μU/mL, and blood pressure in mmHg

^a^Chi-square test is used for statistical evaluation

^b^Mann-Whitney U test is used for statistical evaluation

### Metabolic syndrome components and diagnostic comparisons

Comparisons of metabolic syndrome (MetS) components and diagnostic prevalence between groups are presented in Table 2. Adolescents with obesity exhibited significantly higher rates of elevated waist circumference and serum triglyceride levels compared to controls (*p* < .05 for both). HDL cholesterol levels did not significantly differ between groups.

**Table 2:** Evaluation and comparison of metabolic syndrome components, metabolic syndrome diagnosis, and pediatric siMS scores

	Study group (*n* = 48)	Control group (*n* = 41)	Statistics
IDF criteria for pediatric metabolic syndrome, *n* (%)			
1. High waist circumference	47 (97.9)	9 (22.0)	X^2^ = 54.70, ***p*** **< .01**^**a**^
2. High fasting blood glucose	1 (2.1)	**−**	*p* = 1^b^
3. Low serum HDL	15 (31.3)	7 (17.1)	X^2^ = 2.39, *p* = .12^a^
4. High serum triglycerides	13 (27.1)	3 (7.3)	X^2^ = 5.86, ***p*** **= .02**^**a**^
5. High blood pressure (> 130 − 85 mmHg)	4 (8.3)	2 (4.9)	*p* = .68^b^
IDF Criteria - Metabolic syndrome, n (%)	7 (14.6)	**-**	***p*** **= .01**^**b**^
Expanded criteria for pediatric metabolic syndrome, n (%)			
1. High waist circumference	47 (97.9)	9 (22.0)	X^2^ = 54.70, ***p*** **< .01**^**a**^
2. High fasting blood glucose, hyperinsulinemia, or insulin resistance	20 (41.7)	1 (2.4)	X^2^ = 18.87, ***p*** **< .01**^**a**^
3. Low serum HDL	15 (31.3)	7 (17.1)	X^2^ = 2.39, *p* = .12^a^
4. High serum triglycerides	13 (27.1)	3 (7.3)	X^2^ = 5.86, ***p*** **= .02**^**a**^
5. High blood pressure (> 90. percentile)	21 (43.8)	12 (29.3)	X^2^ = 1.99, *p* = .16^a^
Expanded criteria - Metabolic syndrome, n (%)	21 (43.8)	2 (4.9)	X^2^ = 17.44, ***p*** **< .01**^**a**^
Pediatric siMS scores, median (rank)	2.64 (61.04)	1.79 (26.22)	Z = -6.34, ***p*** **= .00**^**c**^

Statistically significant *p* values are indicated in bold color (*p* < .05)

^a^Chi-square test is used for statistical evaluation

^b^Fisher’s exact test is used for statistical evaluation

^c^Mann-Whitney U test is used for statistical evaluation

None of the control participants met the diagnostic criteria for MetS according to the IDF definition, and only two controls met the threshold under the expanded criteria, consistent with their mild metabolic alterations but absence of obesity.

According to IDF criteria, only one participant in the obesity group exhibited impaired fasting glucose (> 100 mg/dL). However, when using the expanded criteria—which include hyperinsulinemia and insulin resistance—41.7% of adolescents with obesity (*n* = 20) met criteria for impaired glucose metabolism, a rate significantly higher than that of the control group (χ² = 18.87, *p* < .01).

There were no statistically significant group differences in the prevalence of elevated blood pressure using either diagnostic framework. Based on IDF criteria, 14.6% of adolescents with obesity (*n* = 7) met the diagnostic threshold for MetS, significantly higher than the control group (*p* = .01). Using the expanded criteria, 43.8% (*n* = 20) of adolescents in the obesity group were diagnosed with MetS, also significantly higher than controls (*p* < .01).

Furthermore, Pediatric siMS (PsiMS) scores were significantly higher in the obesity group compared to controls (Z = -6.34, *p* < .05) (Table 2).

### Correlations between anthropometric measures and metabolic parameters

Correlation analyses revealed no significant association between fasting glucose and any of the three anthropometric measures (BMI, WC, or NC) in either group.

Fasting insulin levels were positively correlated with BMI in both groups, with significant correlations observed between insulin and WC in the control group, and between insulin and NC in the obesity group (all *p* < .05). Similarly, HOMA-IR values were significantly associated with BMI in both groups, WC in controls, and NC in the obesity group (*p* < .05 for all).

Higher BMI was associated with significantly lower HDL levels in both groups but showed no correlation with serum triglycerides. In contrast, elevated WC and NC were significantly associated with higher triglyceride levels only in adolescents with obesity. Conversely, these same anthropometric measures were linked to lower HDL cholesterol only in the control group (*p* < .05 for all).

In the obesity group, both BMI and NC were positively associated with higher systolic blood pressure, whereas WC was not. Across both groups, higher BMI, WC, and NC values were significantly correlated with an increased number of total MetS criteria according to the IDF definition (*p* < .05). Notably, higher NC was also associated with increased PsiMS scores and a greater number of expanded MetS criteria met—exclusively in the obesity group (*p* < .05). All correlation analyses involving BMI were performed using BMI-for-age percentiles rather than raw BMI values to account for developmental variation during adolescence (Table 3).

**Table 3:** Correlations between anthropometric measurements and metabolic syndrome components

	Body Mass Index		Waist circumference		Neck circumference	
	Study	Control	Study	Control	Study	Control
Fasting blood glucose	0.04	0.14	0.12	0.08	− 0.03	0.01
Serum insulin	**0.34** ^**a**^	**0.53** ^**b**^	0.26	**0.32** ^**a**^	.**36**^**a**^	0.03
HOMA – IR	**0.33** ^**a**^	**0.51** ^**b**^	0.27	**0.31** ^**a**^	**0.32** ^**a**^	0.02
Serum triglycerides	0.25	− 0.06	**0.35** ^**a**^	− 0.04	**0.35** ^**a**^	− 0.24
Serum HDL	**− 0.32** ^**a**^	**− 0.36** ^**a**^	− 0.25	**− 0.45** ^**b**^	− 0.23	**− 0.32** ^**a**^
Systolic BP	**0.29** ^**a**^	0.23	0.23	0.17	**0.32** ^**a**^	0.18
Diastolic BP	0.19	0.08	0.06	− 0.01	0.24	− 0.05
PsiMS score	**0.48** ^**b**^	**0.46** ^**b**^	**0.57** ^**b**^	**0.54** ^**b**^	**0.47** ^**b**^	0.26
IDF total criteria	**0.46** ^**b**^	**0.46** ^**b**^	**0.38** ^**b**^	**0.56** ^**b**^	**0.46** ^**b**^	**0.42** ^**b**^
Exp total criteria	**0.42** ^**b**^	**0.37** ^**a**^	**0.29** ^**a**^	**0.49** ^**b**^	**0.36** ^**a**^	0.28

Statistically significant *p* values are shown in bold color. Correlations for BMI and WC were calculated using age- and sex-adjusted percentile values rather than raw measurements

^a^Spearman’s rho is significant at *p* <.05 level

^b^Spearman’s rho is significant at *p* <.01 level

In multivariate regression models adjusted for age and sex, neck circumference remained independently associated with fasting insulin (β = 0.31, *p* = .02), HOMA–IR (β = 0.29, *p* = .03), and PsiMS score (β = 0.33, *p* = .01), but not with triglycerides or systolic blood pressure (*p* > .05). These findings indicate that NC is an independent anthropometric predictor of insulin resistance and overall metabolic risk in adolescents with obesity.

Receiver operating characteristic (ROC) curve analyses were performed to compare the predictive performance of neck circumference (NC), waist circumference (WC), body mass index (BMI), and BMI percentile for identifying metabolic syndrome. NC demonstrated an area under the curve (AUC) of 0.781 (95% CI: 0.667–0.895, *p* < .001), indicating acceptable discriminatory ability. The predictive performance of NC was comparable to WC (AUC = 0.769), BMI (AUC = 0.758), and BMI percentile (AUC = 0.754) (Table 4).

**Table 4:** ROC curve analysis for predicting metabolic syndrome

Anthropometric parameter	AUC	95% CI	*p*
Neck circumference	0.781	0.667–0.895	< 0.001
Waist circumference	0.769	0.654–0.884	< 0.001
BMI	0.758	0.648–0.867	< 0.001
BMI percentile	0.754	0.632–0.876	< 0.001

**Fig. 1:**
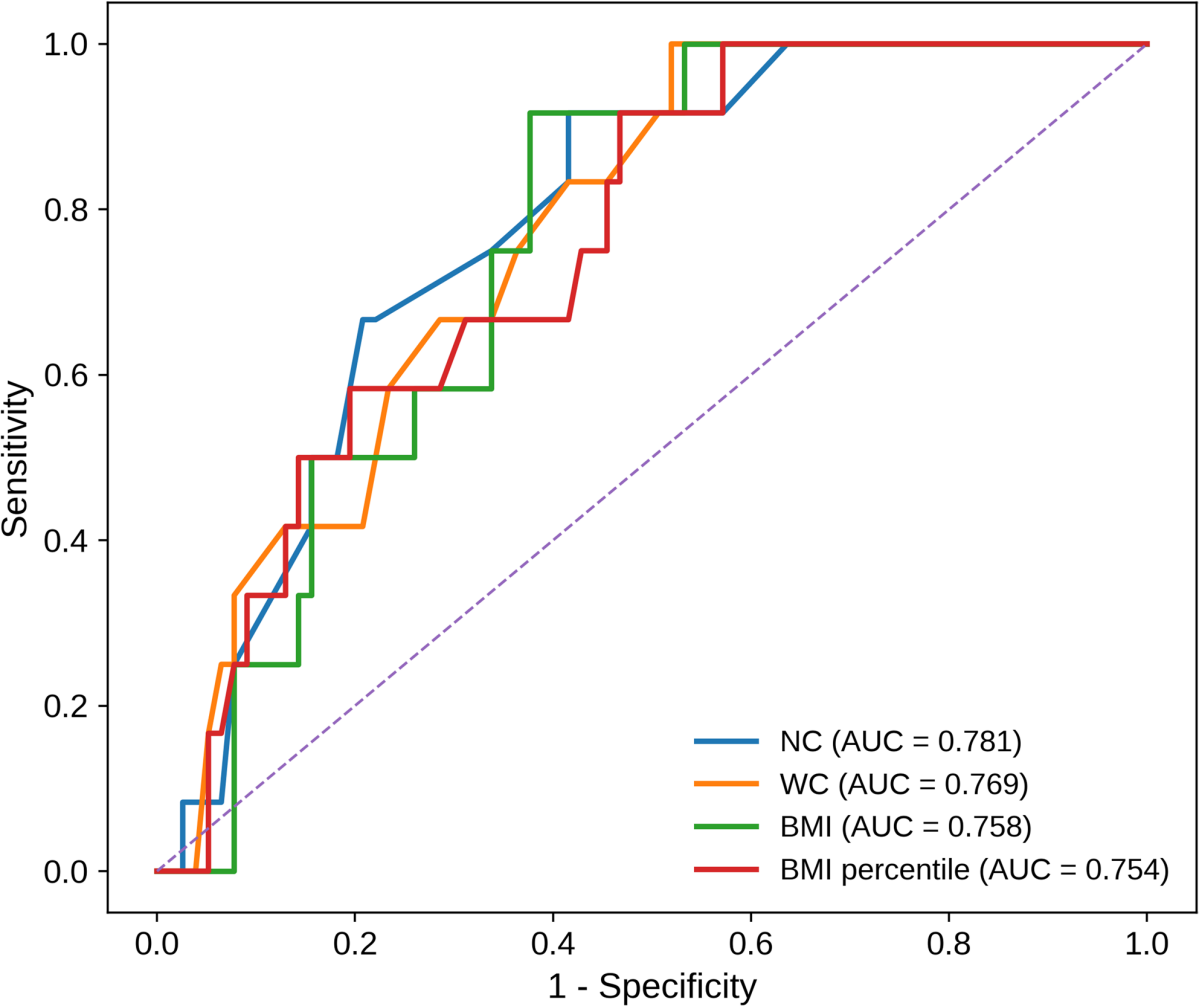
Receiver operating characteristic (ROC) curves comparing the predictive performance of neck circumference (NC), waist circumference (WC), body mass index (BMI), and BMI percentile for identifying metabolic syndrome in adolescents. NC demonstrated the highest area under the curve (AUC = 0.781), followed by WC (0.769), BMI (0.758), and BMI percentile (0.754)

Receiver operating characteristic (ROC) curves comparing the predictive performance of neck circumference, waist circumference, BMI, and BMI percentile are shown in Fig. 1.

## Discussion

Waist circumference (WC) is a widely used anthropometric measure for evaluating central adiposity in both clinical and research settings. However, its variability throughout the day—especially in postprandial states—and its relatively lower specificity in pediatric populations have prompted interest in alternative measures. Neck circumference (NC), in contrast, is easy to perform, less affected by diurnal changes, and has demonstrated moderate diagnostic accuracy in identifying overweight and obesity among children and adolescents. In a meta-analysis including over 11,000 participants aged 6 to 18 years, NC showed a sensitivity of 0.780 and specificity of 0.746 for detecting elevated BMI, with a diagnostic odds ratio of 17.34, supporting its utility as a feasible screening tool in youth populations [6].

In our study, adolescents with obesity had significantly greater NC measurements than their normal-weight peers (mean 37.95 cm vs. 31.86 cm, *p* < .001). This aligns with prior findings and reinforces the value of NC as a practical anthropometric marker for distinguishing adolescents with obesity [29].

Regarding metabolic syndrome (MetS), our findings revealed that adolescents with obesity exhibited significantly higher values across nearly all metabolic parameters compared to controls, except for fasting glucose and diastolic blood pressure. This is consistent with prior literature, including a study by Šimunović et al. which reported that fasting glucose is a relatively weak indicator among MetS components in pediatric populations [30]. While systolic blood pressure was elevated in our obesity group, diastolic pressure did not differ significantly — likely a reflection of our limited sample size rather than a true absence of association.

Notably, 14.6% of adolescents with obesity met MetS criteria based on IDF definitions, and this prevalence rose to 43.8% when expanded criteria were applied—highlighting the sensitivity of broader definitions that include insulin resistance markers such as HOMA-IR and hyperinsulinemia. Moreover, Pediatric siMS (PsiMS) scores were significantly higher among adolescents with obesity, further supporting the role of this tool as a clinically practical surrogate for MetS severity.

Our results echo those of Gomez-Arbelaez et al., who found significant associations between NC and metabolic parameters—including fasting glucose, triglycerides, and blood pressure—in children aged 8–14 years [31]. Discrepancies between our findings and theirs regarding glucose may be attributed to differences in age range or sample size.

Multiple studies in both pediatric and adult populations have supported the association between NC and MetS [18, 31]. The current findings build on this evidence by suggesting that NC may not only serve as a marker of general and central obesity but also function as a potential early indicator of metabolic dysregulation in adolescents.

We observed significant correlations between NC and BMI and WC in participants with obesity and control group, suggesting that NC may reflect overall adiposity across different weight categories. Therefore, it may be worthwhile to incorporate NC measurements into routine screening even in youth without obesity, to aid in early identification of individuals at risk for future obesity and cardiometabolic complications.

A previous Turkish study in pubertal adolescents reported positive associations between NC and all MetS criteria except plasma glucose [19]. Similarly, in our study, NC was significantly associated with fasting insulin levels, HOMA-IR index, serum triglycerides, systolic blood pressure, and mean arterial pressure among adolescents with obesity. These findings are particularly relevant given the emerging role of NC in assessing cardiometabolic risk during puberty. The multivariate analyses adjusting for age and sex confirmed that NC independently predicts insulin resistance and composite metabolic risk, underscoring its potential clinical utility beyond simple correlation.

Interestingly, our analysis did not reveal a consistent association between NC and HDL cholesterol in adolescents with obesity, while a negative association was observed in controls. It is possible that lifestyle factors—such as diet quality or physical activity—may explain the higher HDL levels in the control group; however, the lack of data on lifestyle behaviors limits our ability to draw definitive conclusions. Furthermore, since we did not perform sex-stratified analyses, future research should examine whether these associations vary by gender.

Our study also found a positive correlation between NC and systolic blood pressure in the obesity group, a finding consistent with previous reports in both adolescents and adults [18, 19]. A large case-control study of nearly 2,000 adolescents found that elevated NC, when present alongside abdominal obesity, was significantly associated with elevated blood pressure—suggesting a synergistic risk effect on cardiometabolic risk [32]. Similarly, our results imply that the co-occurrence of increased NC and WC may enhance the predictive power for identifying hypertension risk in adolescents. Larger prospective studies are needed to validate this hypothesis and further elucidate the interaction between NC, abdominal obesity, and cardiovascular risk.

In contrast to previous studies that primarily examined neck circumference (NC) as a marker of general adiposity or as part of sleep apnea risk assessment, our findings add further evidence supporting the association between NC and metabolic risk factors in adolescents, including insulin resistance and the Pediatric siMS score, in a Turkish adolescent population.

By incorporating both standard and expanded definitions of metabolic syndrome, this study demonstrates that NC not only reflects adiposity but also correlates independently with metabolic risk markers such as fasting insulin and HOMA–IR after adjusting for age and sex. These findings highlight the potential value of NC as a practical, low-cost, and non-invasive screening tool for early detection of cardiometabolic risk among adolescents with obesity—particularly in resource-limited clinical settings.

In addition to correlation analyses, ROC curve analyses were conducted to compare the predictive performance of neck circumference with traditional anthropometric indices. The results showed that NC had a predictive performance comparable to BMI and waist circumference in identifying metabolic syndrome. Although NC did not substantially outperform these established measures, it demonstrated slightly higher AUC values and offers practical advantages, as it is simple to measure and less affected by factors such as postprandial abdominal distension or clothing.

### Limitations

This study has several limitations. First, the cross-sectional design precludes any inference of causality between anthropometric indices and cardiometabolic risk. Second, our sample size, although adequate for initial exploratory analysis, limits the statistical power—especially in subgroup comparisons such as sex- or pubertal stage-specific analyses. Third, we did not assess dietary patterns, physical activity, or socioeconomic status, which are important confounders in studies of obesity and metabolic health. Fourth, Although NC values tend to differ slightly between sexes during adolescence, our sample size did not permit detailed sex-stratified analyses or inclusion of sex as a covariate in regression models. Another limitation of our study is that pubertal development (Tanner staging) was not evaluated, which may have influenced anthropometric parameters, particularly neck circumference. However, to mitigate this limitation, age was included as a covariate in the multivariate regression analyses. Nonetheless, as puberty-related hormonal changes may independently affect fat distribution and anthropometric measures, future studies should include Tanner staging or other pubertal maturity indicators to provide more precise adjustment. Finally, our findings are based on a single-center cohort and may not be generalizable to broader or more diverse populations. Future studies with larger cohorts should consider sex-specific reference values and adjustments.

### Conclusion and implications for practice

This study reinforces the clinical relevance of neck circumference as a simple, reliable, and non-invasive tool for identifying adolescents at increased risk for obesity and metabolic syndrome. Beyond its correlation with traditional anthropometric markers, NC was also associated with key metabolic parameters and composite risk scores. Given its practicality and predictive potential, NC measurement may offer a valuable addition to routine pediatric assessments—particularly in resource-limited settings.

Future studies with larger and more diverse populations are warranted to validate these findings, explore longitudinal associations, and determine optimal NC cutoff values by age, sex, and pubertal status. Integrating NC into pediatric screening protocols may facilitate earlier detection of metabolic risk, allowing for timely intervention and prevention of long-term health consequences.

## Data Availability

The data that support the findings of this study are available upon reasonable request.

## Abbreviations

BMI: Body Mass Index
BP: Blood Pressure
DBP: Diastolic Blood Pressure
HDL: High-Density Lipoprotein
HOMA-IR: Homeostasis Model Assessment for Insulin Resistance
IDF: International Diabetes Federation
MetS: Metabolic Syndrome
NC: Neck Circumference
PsiMS: Pediatric Simple Metabolic Syndrome
SBP: Systolic Blood Pressure
WC: Waist Circumference
